# Personalization of prostate cancer prevention and therapy: are clinically qualified biomarkers in the horizon?

**DOI:** 10.1007/s13167-011-0138-2

**Published:** 2012-01-12

**Authors:** Timothy A Yap, Charles Swanton, Johann S de Bono

**Affiliations:** 1 Drug Development Unit, The Royal Marsden NHS Foundation Trust, Downs Road, Sutton, Surrey SM2 5PT, UK; 2 Division of Clinical Studies, The Institute of Cancer Research, 15 Cotswold Rd, Sutton, Surrey SM2 5NG, UK; 3 Translational Cancer Therapeutics Laboratory, Cancer Research UK London Research Institute, 44 Lincoln's Inn Fields, London WC2A 3PX, UK

**Keywords:** Prostate cancer, Intermediate endpoint biomarkers, Novel clinical trial designs, Predictive diagnostics, Targeted prevention, Personalized treatment

## Abstract

Prostate cancer remains the most common malignancy among men and the second leading cause of male cancer-related mortality. Death from this disease is invariably due to resistance to androgen deprivation therapy. Our improved understanding of the biology of prostate cancer has heralded a new era in molecular anticancer drug development, with multiple novel anticancer drugs for castration resistant prostate cancer now entering the clinic. These include the taxane cabazitaxel, the vaccine sipuleucel-T, the CYP17 inhibitor abiraterone, the novel androgen receptor antagonist MDV-3100 and the radionuclide alpharadin. The management and therapeutic landscape of prostate cancer has now been transformed with this growing armamentarium of effective antitumor agents. This review discusses strategies for the prevention and personalization of prostate cancer therapy, with a focus on the development of predictive and intermediate endpoint biomarkers, as well as novel clinical trial designs that will be crucial for the optimal development of such anticancer therapeutics.

## Introduction

Prostate cancer is the leading malignancy among males and the second most common cause of male cancer-related deaths after lung cancer [1]. Although the disease is potentially curable with local therapy when confined to the prostate gland, approximately 33% of patients develop resistance to local treatments and eventually progress to have incurable metastatic disease. Mortality from this disease is usually due to resistance to androgen deprivation therapy and the eventual development of castration resistant prostate cancer (CPRC). The molecular etiology of the development of resistance to prostate cancer therapies is still not fully understood, with previous and ongoing studies hindered by inadequate preclinical models and issues in acquiring prostate tumor tissue [2].

## Novel strategies in castration resistant prostate cancer

Strategies developed to counteract androgen-deprivation therapy resistance have had only modest clinical benefit. Indeed, prior to 2010, only docetaxel chemotherapy improved overall survival in patients with CRPC compared with mitoxantrone [3]. However, with recent developments in novel chemotherapeutics and targeted agents, there appears to be a new dawn in the management of prostate cancer, with a number of novel anticancer drugs for CRPC recently entering the clinic. The key antitumor agents that have shown greatest promise include the novel taxane cabazitaxel (*Jevtana*, Sanofi-Aventis) [4], the vaccine sipuleucel-T (Provenge; Dendreon) [5], the CYP17 inhibitor abiraterone (Zytiga; Ortho Biotech) [6], the novel androgen-receptor antagonist MDV-3100 (Medivation/Astellas) [7] and the radioisotope alpharadin (radium 223; Algeta/Bayer Pharma AG) (Table 1) [3].

**Table 1:** Key clinical trials in castration resistant prostate cancer.

Agent	Phase	No. of patients	PSA RR >50%	Median OS (months)	Median PFS (months)	Comments
Prednisone + docetaxel vs docetaxel vs mitoxantrone + prednisone	III	1,006 (CT-naive)	45% vs 48% vs 32%	18.9 vs 17.4 vs 16.5	NA	Docetaxel approved as first line therapy for CRPC
Sipuleucel-T vs placebo	III	512 (CT-naive)	2.6% vs 1.3%	25.8 vs 21.7	3.7 vs 3.6 (TTrP)	Sipuleucel-T approved for CT-naive patients with asymptomatic or mildly symptomatic CRPC
Prednisone + abiraterone vs prednisone + placebo	III	1,195 (post-CT)	29.1% vs 5.5%	14.8 vs 10.9	5.6 vs 3.6	Abiraterone approved for post-docetaxel setting
Prednisone + cabazitaxel vs prednisone + mitoxantrone	III	755 (post-CT)	39.2% vs 17.8% (overall PSA RR)	15.1 vs 12.7	2.8 vs 1.4	Cabazitaxel new standard second line chemotherapy
MDV3100 vs placebo	III	1199 (Interim analysis triggered at 520 events)	NA	18.4 vs 13.6 (37% reduction in risk of death)	NA	Interim analysis
Alpharadin vs placebo	III	922	NA	14 vs 11.2	NA	Interim analysis

Prior to 2010, only docetaxel chemotherapy demonstrated a survival benefit in patients with CRPC compared with mitoxantrone. Since then, sipuleucel-T, abiraterone, cabazitaxel, MDV3100 and alpharadin have met their respective primary endpoints in phase III clinical trials. These novel agents have now obtained regulatory approval, or are expected to be approved in due course

*CRPC *castration-resistant prostate cancer; *CT *chemotherapy, *NA *not available; *OS *overall survival; *PFS *progression-free survival; *PSA *prostate-specific antigen; *RR *response rate; *TTP *time to progression; *TTrP *time to radiological progression

It is therefore now critical that these novel agents are appropriately applied to the CRPC treatment pathway to maximize benefit for patients suffering from advanced-stage prostate cancer, while minimizing toxicities and cost. Thus, the discovery of biomarkers and diagnostics for prostate cancer screening, as well as the development of predictive and intermediate endpoint biomarkers and novel clinical trial designs will be crucial for the optimization of novel molecularly targeted therapeutics in CRPC [3].

This review discusses strategies for the prevention and personalization of prostate cancer therapy, with a focus on the development of predictive and intermediate endpoint biomarkers, as well as novel clinical trial designs that will be crucial for the optimal development of such anticancer therapeutics.

## Screening strategies in prostate cancer

The detection rate and incidence of prostate cancer has risen in recent decades partly because of the increased use of prostate specific antigen (PSA) as a screening tool [8]. This has led to an increased pick up rate in localized prostate cancers, most of which comprise low grade and low volume prostate tumors. Historically, autopsy studies have shown that incidental prostate cancer is relatively common, especially in older males. Thompson and colleagues estimated the prevalence of prostate cancer in males aged between 55 and 70 years to be approximately 6% and following 7 years of surveillance, prostate cancer was diagnosed in 24% of males in the control arm, and 18% of males in the treatment arm [9]. Whilst all prostate cancers will inevitably progress, many do so at relatively slow rates and never actually lead to clinically significant consequences. As such, the risk-benefit balance in the early detection and intervention of prostate cancer has to be considered in such patients. The risks of peri-operative complications, long term issues such as urinary incontinence and impotence need to be considered. In contrast, males with aggressive prostate malignancies will benefit from an active surveillance approach. As a result of this great variation in the potential aggressiveness of prostate cancer and the non-cancer specific nature of PSA, great debate has ensued on the appropriateness of PSA screening.

### Prostate cancer screening trials

Two recent large randomized clinical trials published in the New England Journal of Medicine on prostate cancer screening demonstrated that prostate cancer related deaths are relatively infrequent during the first 10 years following screen detection and that up to 48 males have to be identified in order to prevent a single prostate cancer death [10,11]. While the U.S. trial showed no benefit in mortality from screening, the European study demonstrated that a small decline in prostate cancer mortality with large numbers of subjects required to receive aggressive treatments to benefit a relative few. Significantly, it has been estimated that up to US$5.2 million would need to be spent on screening and interventions, to prevent a single prostate cancer-related death.

### U.S. Preventative Services Task Force guidelines

Following these data and evidence from other key clinical trials, the U.S. Preventative Services Task Force (USPSTF) recently released guidelines advising against PSA-based screening for prostate cancer in asymptomatic men [12]. The USPSTF acknowledged that there are data supporting the detection of many cases of asymptomatic prostate cancer through PSA-based screening. Although the majority of such PSA-screen detected asymptomatic individuals have a tumor that meets histological criteria for prostate cancer, the tumor will either not progress or is so indolent, it will not impact mortality. In view of this, better biomarkers and novel diagnostics are clearly required for the detection of potentially aggressive prostate cancer.

## A new era in CRPC antitumor therapeutics

Our improved understanding of the underlying biology of CRPC has heralded a new era in molecular anticancer drug development, with a number of novel anticancer drugs for CRPC recently entering the clinic (Table 1). It is thus now critical that agents such as abiraterone [6], MDV-3100 [7], cabazitaxel [4], sipuleucel-T [5], and alpharadin [3] are appropriately applied to the CRPC treatment pathway to maximize benefits for patients suffering from CRPC. Thus, the development of predictive and intermediate endpoint biomarkers, and the incorporation of novel clinical trial designs are crucial for the optimal development of such anticancer targeted therapies [3].

## Predictive biomarkers in prostate cancer

The rational selection of appropriate populations of patients for molecular therapeutics is important in a heterogeneous disease like CRPC, in order to fine tune antitumor responses to selective targeted therapeutics. We envision that the use of predictive and enrichment biomarkers will be essential to the optimal development of targeted therapeutics. An often cited example is tumoral *TMPRSS2*-*ETS *gene fusion in CRPC, which may, in the future, prove to predict for antitumor responses to novel agents, such as abiraterone [13].

### TMPRSS2-ETS gene fusion in CRPC

The ERG gene has been observed to be one of the most commonly overexpressed proto-oncogenes in prostate cancer and present in approximately 72% of cases of prostate cancer [14]. Tomlins and colleagues demonstrated that TMPRSS2, a serine protease secreted by prostate epithelial cells in response to androgen exposure, is fused to ERG, a member of the ETS family of oncogenes [15]. This rearrangement results in the creation of a fusion transcript called TMPRSS2-ETS. This leads to the overexpression of the ETS oncogene, initially under androgen and the androgen receptor (AR) control, although androgen dependence may be eventually lost in advanced disease. It now appears that the activation of this pathway may be central to prostate oncogenesis [16,17]. Recent clinical studies assessing abiraterone have indicated that the presence of an *ERG *rearrangement was associated with the magnitude of PSA decline following abiraterone treatment (*P *= 0.007) [13]. This association is now being evaluated prospectively in phase III CPRC clinical trials and results are eagerly anticipated.

## Intermediate endpoint biomarkers in prostate cancer

Another major issue that needs to be urgently addressed for the personalization of prostate cancer is the discovery and development of analytically validated and clinically qualified intermediate endpoint or surrogate biomarkers of clinical benefit [3].

### Urgent need for qualified intermediate endpoint biomarkers in CRPC

Several aspects of the clinical behavior of prostate cancer beggar the need for intermediate endpoint biomarkers in late phase clinical trials. Firstly, the measurement of the primary tumor within the prostate can be inaccurate with conventional imaging modalities. In addition, prostate cancer frequently results in bone metastases, rather than visceral disease that may be easily and accurately measured using conventional radiological methods. In contrast, bony disease is difficult to quantify and the assessment of existing lesions on bone scans is unreliable.

Although a proportion of patients do have measurable disease, and while trials may indeed be limited to this patient population, the presence of measurable visceral disease is usually associated with a more aggressive phenotype and therefore worse prognosis. Therefore, selecting patients with measurable disease for these trials may lead to biased patient selection and restrict the extrapolation of the trial data to the general prostate cancer population. Tumor measurements are therefore difficult to utilize as a predictor of clinical benefit in clinical trials of this disease.

### PSA is not a validated intermediate endpoint biomarker

PSA is not specific to prostate cancer and may be present in million-fold higher concentrations in prostatic secretions, with PSA leakage potentially altering measured levels in patients. In addition, it is now accepted that PSA gene expression is due to AR transcriptional activity, and therefore altered AR transcription could lead to declines in PSA levels, but not in the desired reduction in tumor growth.

While PSA progression has been shown to be significantly associated with survival, changes in PSA are not robust intermediate endpoint biomarkers of survival. This was demonstrated in the three-arm TAX-327 trial of three-weekly or weekly docetaxel, both with prednisone, versus mitoxantrone and prednisone in patients with advanced CRPC [18]. This study demonstrated a survival advantage for 3-weekly docetaxel over mitoxantrone (*p *< 0.01), with no significant survival advantage for the weekly docetaxel arm. However, both docetaxel schedules resulted in similar rates of PSA decrement rates (45% and 48% of patients had a PSA fall >50% on the 3-weekly and weekly schedules respectively, versus 32% on the mitoxantrone arm). Based on these data, it was concluded that PSA declines by >50% are not a surrogate biomarker for overall survival or patient benefit.

Retrospective analyses of data from several randomized clinical trials with the anti-androgen bicalutamide also report that PSA is not a valid intermediate endpoint biomarker in advanced prostate cancer [19]. These data indicate that overall survival and quality of life remain the optimal approvable primary endpoints for such studies [20]. For patients with progressive prostate cancer and castrate levels of testosterone, the Prostate Cancer Clinical Trials Working Group (PCWG2) recommends an increased emphasis on time-to-event endpoints, such as failure to progress, as decision aids in moving from phase II to III clinical trials [21].

It is important to note that progression-free survival (PFS) is a poor intermediate endpoint of overall survival in prostate cancer, with overall associations between PFS and overall survival at best moderate (0.4 for radiographic PFS and 0.33 for PSA PFS) [21].

### Clinical trial endpoints in prostate cancer

Due to the necessity to measure overall survival as a primary endpoint, Phase III clinical trials evaluating novel drugs in prostate cancer have traditionally involved large patient cohorts and an extended period of follow-up. This has led to delays in the analysis and reporting of trial data and the availability of level I evidence that could potentially improve patient care. Replacing overall survival (true endpoint) with an intermediate endpoint that accurately reflects the benefit of new treatments may potentially accelerate drug development [22]. Such surrogate biomarkers could be assessed earlier and more frequently to accelerate treatment advances. This necessity to expedite oncological drug development is reflected by changes in cancer drug approval regulations. These regulations now allow accelerated approval when clinical trials establish that the new antitumor agent has an effect on a surrogate endpoint that is "reasonably likely" to predict clinical benefit on the basis of an effect on an endpoint other than survival.

These regulatory changes reflect increasing public pressure for the rapid approval of promising new molecularly targeted therapies generated by our burgeoning knowledge of the molecular biology of prostate cancer. The urgent need to develop novel endpoints for prostate cancer trials led the US Food and Drug Administration to conduct a public workshop in 2004 on clinical trial endpoints in prostate cancer. At this workshop, endpoints regarded as acceptable for the regulatory approval of new drugs in prostate cancer were discussed [23]. These discussions indicated that novel validated biomarkers, established as "true" surrogates of clinical benefit, are urgently needed for the management of patients with advanced prostate cancer.

With several antitumor agents recently approved for use in CRPC and numerous others in late phase clinical trials, it is likely to be increasingly more difficult to demonstrate a survival benefit for future drugs since crossover to an effective treatment may confound an overall survival effect [3,24]. This may lead to larger, more costly and protracted studies [3]. There is thus an urgent unmet need in CRPC for an intermediate endpoint biomarker for survival, especially since conventional methods, e.g. bone scan changes are difficult to interpret and changes in PSA levels are not surrogates for survival [21].

Since the measurement of PSA levels is not a validated surrogate biomarker of antitumor response, there is an urgent need for the development of novel intermediate endpoint biomarkers; this may potentially include the enumeration of circulating tumor cells (CTCs).

## Personalizing prostate cancer treatments with circulating tumor cells

Changes in CTC counts from baseline during treatment are now recognized as a prognostic marker in patients with metastatic breast, prostate and colorectal cancer [25-27]. This validated enumeration is feasible due to advances in technology that allow the automated and high-throughput separation, visualisation and quantitation of cancer cells from blood [28]. The study of CTC with the CellTracks^® ^system (Immunicon, PA), allows highly reproducible (85-95% reproducibility between laboratories) enumeration of CTCs [28]. CTCs are not detected in healthy subjects or in patients with non-malignant diseases. Although CTC counts are of prognostic relevance, CTC enumeration has not been validated as a surrogate of clinical benefit.

The ability to longitudinally evaluate gene amplifications, mutations, deletions or translocations that have crucial roles in CRPC pathogenesis with CTCs provides unique insights into the underlying and evolving biology of the tumor, without the necessity for invasive biopsies [29]. This will also allow the analysis of molecular changes that occur secondary to treatment pressures and intra-patient tumor heterogeneity that may otherwise have been missed with tumor biopsies [29]. It also allows patient stratification according to the molecular profiles of risk, prognosis and likely response.

### CTC enumeration as an intermediate endpoint biomarker

Highly significant differences in survival based on changes in CTC counts following cytotoxic and hormone therapy have been observed. The IMMC38 trial evaluated the impact of cytotoxic chemotherapy on CTCs and PSA in patients with CRPC. Significantly, the fold change of CTCs at 4, 8 and 12 weeks following treatment was the most important prognostic factor in multivariate analyses (*p *< 0.0001), suggesting that CTC count declines were able to identify patients likely to benefit from cytotoxic chemotherapy [13,26,30,31]. In contrast, PSA declines added little benefit to the performance of the receiver-operator curve. These data have now been validated prospectively in the randomized Phase III abiraterone trial [6] where CTC count declines at 4, 8 and 12 weeks after starting therapy met the statistical requirements for surrogacy of overall survival [32]. These CTC analyses in almost 1,000 patients demonstrated that both CTC baseline values were highly concordant (*r *= 0.83) and CTC declines and conversion (from CTC ≥ 5 to < 5) were strongly prognostic as early as 4 weeks after treatment, with abiraterone resulting in a highly significant increased likelihood of falling CTC counts. In addition, the introduction of CTC count declines into a multivariate prognostic model reduced the treatment effect significantly (HR: 0.74 to 0.97), indicating that CTC count declines explained much of the treatment effect on survival and meet stringent surrogacy criteria [32].

## Clinical trial designs in CRPC

Designing clinical trials for CRPC remains challenging because of a number reasons; the standard criteria for measuring radiological responses - Response Evaluation Criteria In Solid Tumors (RECIST) - is not always applicable because measurable disease occurs infrequently in patients with CRPC. In addition, the natural history of this disease may potentially be sustained over decades because of the slow rate of growth of certain prostate cancers and finally because the target population is mainly elderly and possibly more fragile and sensitive to treatment-related toxicities. Thus, the design of clinical trials in CRPC is a constantly evolving paradigm, especially with the development of novel anticancer therapies, including both molecularly targeted drugs and immunotherapies.

### Prostate Cancer Clinical Trials Working Group guidelines

In 2008, a committee of investigators defined new consensus criteria to plan and conduct trials for prostate cancer [21]. The PCWG2 developed guidelines to conduct clinical trials for patients with CRPC, standardizing trial endpoints for agents that act through diverse mechanisms. The PCWG2 distinguished two types of study efficacy objectives: the control, relief, or elimination of disease manifestations (e.g. pain) that are present when treatment is initiated, and the prevention or delay of disease manifestations (e.g. death) expected to occur. The PCWG2 recommended an increased emphasis on time-to-event endpoints (i.e. failure to progress) as decision aids in proceeding from phase II to phase III trials, as well as the reporting of outcomes independently for PSA, imaging, and clinical measures, avoiding grouped categorizations such as complete or partial response. Early changes in PSA and/or pain should not be acted on without other evidence of disease progression, and treatment should be continued for at least 3 months to ensure adequate drug exposure. Bone scans should be reported as "new lesions" or "no new lesions," changes in soft-tissue disease assessed by RECIST, and pain using validated scales. These PCWG2 recommendations are due to evolve as data are generated on the utility of intermediate end points to predict clinical benefit.

### Adaptive clinical trial strategies

In view of the unique features of CRPC, customized, adaptive, hypothesis-testing trial designs should be considered in future clinical trials. Such studies may permit the alteration of the randomization rate during a trial in real time to increase the chance that a patient is allocated to the best treatment. Adaptive trial strategies permit the statistical model to learn as the study progresses through interim assessments by adapting sample size or discontinuing the trial early for success, futility or harm. Also, such studies may allow the switching of hypothesis of non-inferiority to superiority or vice-versa, or dropping of selected arms or doses. Recent examples of clinical studies using an adaptive strategy are the BATTLE trial in non-small cell lung cancer [33] and I-SPY2 study [34] in locally advanced breast cancer.

## Future outlook and conclusions

The management and therapeutic landscape of prostate cancer is clearly changing with the recent approval of a range of effective antitumor agents. With the armamentarium of drugs available for routine use in advanced prostate cancer, it is critical that they are now applied appropriately in order to maximize patient benefit and minimize costs. Potential issues including drug-related toxicities and cross-resistance to individual agents after exposure to a prior treatment should be considered. In the future, it is likely that transition from one treatment regimen to the next should be based on a combination of clinical, biochemical and radiological factors. Importantly, novel biomarker assays, such as validated predictive and intermediate endpoint biomarkers will need to be routinely incorporated into treatment pathways in the future. Much work also needs to be continued in the future for the development of robust diagnostics for prostate cancer screening.
